# The Excessive “Heated Terms”

**Published:** 1882-07

**Authors:** 


THE EXCESSIVE "HEATED TERMS" affected the composition of the ink-rollers on our printing press so that the fluid was not regulaıly distributed on the type, which caused bad press-work in some of the copies of the June issue. This uncontrollable misfortune we hope our readers will overlook. We trust that we will not again have another such spell of weather, not only for the safety of our ink-rollers, but for human life, the mortality of which has been unusual in our city during these "heated terms."

